# Association of Residual Plasma Viremia and Intima-Media Thickness in Antiretroviral-Treated Patients with Controlled Human Immunodeficiency Virus Infection

**DOI:** 10.1371/journal.pone.0113876

**Published:** 2014-11-21

**Authors:** Anders Boyd, Jean-Luc Meynard, Laurence Morand-Joubert, Adrien Michon, Franck Boccara, Jean-Philippe Bastard, Assia Samri, Nabila Haddour, Ziad Mallat, Jacqueline Capeau, Moïse Desvarieux, Pierre-Marie Girard, for the Collaboration in HIV, Inflammation and Cardiovascular Disease Study

**Affiliations:** 1 INSERM UMR_S 1136, Institut Pierre Louis d′Epidémiologie et de Santé Publique, Paris, France; 2 Sorbonne Université, UPMC Univ Paris-6, Paris, France; 3 Department of Infectious and Tropical Diseases, Hôpital Saint-Antoine, AP-HP, Paris, France; 4 Sorbonne Universités, UPMC Univ Paris 06, INSERM, UMR_S 1136, Institut Pierre Louis d′Epidémiologie et de Santé Publique, Laboratoire de Virologie, Saint Antoine, APHP, Paris, France; 5 Service de médecine interne, Hôpital Européen Georges Pompidou, AP-HP, Paris, France; 6 Department of Cardiology, Hôpital Saint-Antoine, AP-HP, Paris, France; 7 INSERM UMR_S 938, Paris, France; 8 APHP, Hôpital Tenon, Service de biochimie et hormonologie, Inserm UMR_S938, and Institute of Cardiometabolism and Nutrition, Paris, France; 9 Inserm, UMR-S945, IFR113, Department of Immunology, Paris, France; 10 Inserm U970, Cardiovascular Research Center, and Université Paris-Descartes University, Paris, France; 11 Department of Medicine, University of Cambridge, Cambridge, United Kingdom; 12 Department of Epidemiology, Columbia University, Mailman School of Public Health, New York, New York, United States of America; 13 Inserm U738 and Ecole des Hautes Études en Santé Publique, Paris, France; University of Pittsburgh Center for Vaccine Research, United States of America

## Abstract

**Background:**

While residual plasma viremia is commonly observed in HIV-infected patients undergoing antiretroviral treatment (ART), little is known about its subclinical consequences.

**Methods:**

This cross-sectional study included 47 male, never-smoking, non-diabetic patients with ≥4 years of ART and controlled HIV-replication (HIV-viral load, VL <20 copies/mL for ≥1 year). Residual HIV-VL was measured using an ultrasensitive assay (quantification limit: 1 copy/ml). Patients were categorized as having detectable (D; 1-20 copies/mL, *n* = 14) or undetectable (UD; <1 copies/mL, *n* = 33) HIV-VL. Linear regression was used to model the difference in total carotid intima-media thickness [c-IMT, measures averaged across common carotid artery (cca), bifurcation, and internal carotid artery] and cca-IMT alone across detection groups. Multivariable models were constructed for each endpoint in a forward-stepwise approach.

**Results:**

No significant differences were observed between viremia groups with respect to median ART-duration (9.6 years, IQR = 6.8–10.9), nadir CD4+T-cell (208/mm^3^, IQR = 143–378), and CD4+T-cell count (555/mm^3^, IQR = 458–707). Median adjusted inflammatory markers tended to be higher in patients with D- than UD-viremia, with differences in IL-10 being significant (*p* = 0.03). After adjustment on age, systolic blood pressure, and insulin resistance, mean cca-IMT was significantly lower in patients with undetectable (0.668 mm±0.010) versus detectable viremia (0.727 mm±0.015, *p* = 0.002). Cca-IMT was also independently associated with age and insulin resistance. Mean adjusted total c-IMT was no different between viremia groups (*p* = 0.2), however there was large variability in bifurcation c-IMT measurements.

**Conclusions:**

Higher cca-IMT was observed in patients with detectable, compared to undetectable, HIV-VL in never-smoking ART-controlled patients, suggesting that residual HIV viremia may be linked to atherosclerosis.

## Introduction

Among HIV-1-infected patients, antiretroviral therapy (ART) typically leads to the suppression of HIV-replication below the quantification limit of many conventional techniques. Even though these patients would be considered as having controlled HIV viral load (usually at <50 copies/mL), roughly two-thirds continue to exhibit residual viremia between 1–50 copies/mL during long-term ART. [1]–[4] The consequences of residual viremia are poorly understood and while several recent studies have examined the effect of residual viremia on various inflammatory markers [3], [5], these findings have rarely extended to the subclinical or clinical level. [6]

We have previously reported a strong association between increased carotid intima media thickness (c-IMT) and known HIV-duration, regardless of ART, implying that c-IMT could still be increasing during infection despite control of HIV-replication. [7] Residual viremia and alteration in inflammatory cytokines and/or chemokines could be potential explanations for this observation. We thus aimed to compare a number of pro-inflammatory and anti-inflammatory markers, chemokines, and c-IMT in an antiretroviral-treated population of non-smoking, male patients according to levels of residual viremia.

## Materials and Methods

### Study design

The Collaboration on HIV, Inflammation and Cardiovascular Disease study was specifically designed to study the impact of treatment versus HIV on subclinical vascular disease. [7] Inclusion criteria were heavily restricted as to reduce confounding by specifically smoking and gender. Briefly, fifty age-matched (±5 years) triads (*n* = 150) of never-smoking, male subjects were enrolled in three groups: a) HIV-infected patients >35 years old, undergoing ART for ≥4 years, and with HIV-1 RNA viral load <400 copies/mL; b) HIV-infected patients for ≥2 years, ART-naïve and not meeting the indication for ART-initiation; and c) HIV-negative as confirmed by serology. All patients were recruited from Saint-Antoine Hospital (Paris, France) between March 2008-June 2009 and provided written informed consent. The protocol was approved by the Hôtel-Dieu Hospital Ethics Committee (Paris, France).

For this sub-study, we decided to primarily focus on ART-treated patients with undetectable HIV replication based on standard techniques. Only the 50 ART-treated patients from group (a) were therefore included. Among them, three patients were additionally excluded because they had an HIV-VL between 20-400 copies/mL. In total, 47 patients were included in present analysis.

### Quantification of biochemical and virological parameters

HIV-VL was measured using an adapted Cobas AmpliPrep/Cobas TaqMan HIV-1 assay (Roche Diagnostics, Meylan, France; quantification limit: 1 copy/ml). [7] Patients were defined as having either detectable (D; 1–20 copies/mL, *n* = 14) or undetectable (UD; <1 copies/mL, *n* = 33) levels of HIV-viremia.

Inflammatory markers and chemokines were selected for their specific role in HIV-infection [7] and quantified from serum samples stored at −80°C. Interleukin (IL)-6, IL-18, IL-10, IL-27 (Bender Medsystems, Burlingame, CA, USA), resistin (R&D Systems, Minneapolis, MN, USA), total and high molecular weight (HMW) adiponectin (Bühlman, Düsseldorf, Germany) levels were analyzed using an enzyme-linked immunosorbent assay. Serum high-sensitivity C-reactive protein (hs-CRP) and serum amyloid-A (SSA) were measured by immononephelometry on an IMMAGE analyzer (Beckman-Coulter, Miami, FL, USA). Plasma D-dimer was measured by enzyme linked fluorescent assay on a VIDAS analyzer (Biomérieux, Marcy-l′Etoile, France). Chemokines were quantified from plasma using the BD Cytometric Bead Array system (BD, Franklin Lakes, NJ, USA).

### Assessing c-IMT and hypertension

c-IMT scanning and reading protocols have been detailed elsewhere. [7], [8] Total c-IMT was calculated as a composite measure (mean of 12 sites), combining near and far wall of the common carotid artery (cca)-IMT, bifurcation c-IMT, and internal carotid artery (ica)-IMT, bilaterally. Measures with the highest variability were bifurcation c-IMT (SD = 0.106, 75^th^-25^th^%tile = 0.130), followed by cca-IMT (SD = 0.082, 75^th^-25^th^%tile = 0.093) and ica-IMT (SD = 0.071, 75^th^-25^th^%tile = 0.100). Hypertension was defined by prior physician′s diagnosis, systolic blood pressure ≥140 mmHg, diastolic blood pressure ≥90 mmHg, or the use of antihypertensive drugs.

### Statistical analysis

In order to be consistent with previous analyses [7], median pro-/anti-inflammatory markers and chemokines were adjusted *a priori* by age, hypertension, and duration of ART using quantile regression. Adjusted median levels were compared between D and UD groups using a two-tailed t-test based on estimated variance. [9]

Linear regression was used to model the mean difference in total c-IMT and cca-IMT levels comparing D versus UD groups (Δ). Univariate models were constructed to estimate the mean difference in total c-IMT and cca-IMT levels across various risk-factors. A multivariable model was constructed using a predictive approach, while forcing viremia group in the model and including risk-factors with a *p*<0.05 from univariate analyses in a forward-stepwise manner. In order to avoid model overfitting, we evaluated the addition of each covariable using the Bayesian Information Criteria, which penalizes model complexity by the number of observations. After fitting the full multivariable model, other HIV and metabolic risk factors were added individually to the full model so that residual confounding could be assessed. Multivariable models were also constructed for the bifurcation c-IMT and ica-IMT segments, however residual confounding was not assessed. All statistics were performed using STATA (v12.1, College Station, TX, USA).

## Results

Patient characteristics are described in Table 1, while stratified on viremia group. Patients were at low-risk of cardiovascular disease (CVD) [median (IQR) D:A:D score [10] of 10-year CVD-risk: 3.2% (2.6%–5.6%)], while three patients had concomitant statin-use. Three patients had BMI >30 kg/m^2^ and no patients were diagnosed with glucose intolerance or diabetes.

**Table 1:** Demographic and HIV characteristics between viremia groups.

	D-viremia	UD-viremia	
	(*n* = 14)	(*n* = 33)	p*
**General characteristics**			
Age, years†	41 (37–48)	40 (37–43)	0.2
BMI, kg/m^2^ †	23 (21–24)	22 (21–25)	0.8
Hypertension**	2 (14.3)	1 (3.0)	0.2
Total cholesterol *mmol/L* ††	5.05 (4.48–5.28)	4.68 (4.21–5.70)	0.5
HDL cholesterol *mmol/L* ††	1.20 (0.79–1.37)	1.09 (0.95–1.31)	0.4
LDL cholesterol *mmol/L* ††	3.10 (2.74–3.29)	3.19 (2.60–3.91)	0.8
Triglycerides *mmol/L* ††	1.63 (0.81–2.44)	1.19 (0.92–2.03)	0.3
Fasting glucose *mmol/L* ††	4.52 (4.07–5.00)	4.53 (4.28–4.97)	0.9
HOMA-IR††	1.69 (1.20–2.33)	1.41 (1.07–1.91)	0.2
QUICKI††	0.355 (0.344–0.371)	0.365 (0.350–0.384)	0.5
**HIV Infection**			
Duration of HIV infection, years†	13.0 (7.9–17.0)	10.4 (7.6–15.1)	0.5
Duration of ART, years†	10.5 (5.2–11.4)	9.5 (7.1–10.5)	0.8
CD4+ count, cells/mm3†	475 (420–771)	557 (508–703)	0.5
Nadir CD4+ count†	147 (88–364)	254 (170–378)	0.2
CD8:CD4 ratio†	1.20 (0.91–1.57)	1.31 (0.89–1.57)	0.9
HIV-RNA, copies/mL†	6 (3–10)	0	*ntp*
Years from last HIV-VL >50†	3.4 (1.4–5.0)	4.1 (2.1–6.3)	0.5
	D-viremia	UD-viremia	
	(*n* = 14)	(*n* = 33)	p*
**Antiretroviral medication**			
Treatment with NRTI**	13 (92.9)	32 (97.0)	0.5
Duration of NRTI treatment†	147.9 (91.7–199.6)	124.7 (79.1–193.1)	0.4
Treatment with NNRTI**	6 (42.9)	17 (51.5)	0.6
Duration of NNRTI treatment†	67.1 (18.5–109.7)	100.4 (81.4–115.6)	0.3
Treatment with PI**	9 (64.3)	12 (36.4)	0.1
Duration of PI treatment†	107.3 (95.9–127.9)	77.1 (59.7–134.0)	0.3
Treatment with raltegravir**	2 (14.3)	0	0.08

All patients were male, non smokers.

*Significance determined using a Kruskal-Wallis equality-of-populations rank test for continuous variables and Pearson χ^2^ test or Fisher's Exact test for categorical variables;

**number (%);

^†^median (25-75^th^%tile).

^†^^†^Medians (25th and 75th %tiles) adjusted by age, hypertension, and duration of ART using quantile regression. Significance determined using a two-tailed t-test based on variance estimations.

D – detectable; UD – undetectable; HDL – high-density lipid; LDL – low-density lipid; HOMA-IR – homeostatic model assessment of insulin resistance; QUICKI – quantitative insulin sensitivity check index; HIV – human immunodeficiency virus; ART – antiretroviral therapy; cp/mL – copies/mL; VL – viral load; NRTI – nucleos(-t)ide reverse transcriptase inhibitor; NNRTI – non-nucleos(-t)ide reverse transcriptase inhibitor; PI – protease inhibitor; *ntp* – no test performed.

### Inflammatory markers and residual-viremia

Median adjusted pro-inflammatory markers tended to be higher in patients with D- than UD-viremia (hs-CRP: 3.11 vs 2.43 mg/l; IL-18: 213.8 vs 89.7 pg/mL; resistin: 6.48 vs 6.25 ng/ml; IL-6: 1.33 vs 0.79 pg/mL; D-Dimer: 147.4 vs 139.3 ng/ml, respectively) as was insulinemia (7.3 vs 6.0 mU/l), although no significant differences were observed (Table 2). Likewise, patients with D-viremia exhibited higher adjusted median levels of anti-inflammatory markers than with UD-viremia (HMW/total adiponectin: 1.06/3.61 vs 0.92/2.54 µg/ml; IL-27: 1325 vs 1111 pg/mL; IL-10: 0.94 vs 0.65 pg/mL, respectively), with only differences in IL-10 being significant (*p* = 0.03). In addition, no significant difference in chemokines were observed between groups, yet Mig was roughly 1.5-times higher and fractalkine 2-times higher in patients with D- vs. UD-viremia.

**Table 2:** Description of pro- and anti-inflammatory markers between viremia groups.

		D-viremia	UD-viremia
	(*n* = 14)	(*n* = 33)	p*
**Pro-inflammatory**			
Hs-CRP *mg/l*	3.11 (0.59–4.82)	2.43 (0.65–5.14)	0.5
Resistin *ng/ml*	6.48 (5.06–8.06)	6.25 (4.40–8.66)	0.9
IL-18** *pg/mL*	213.8 (134.3–305.7)	89.7 (4.5–180.3)	0.06
IL-6** *pg/mL*	1.33 (0.79–1.57)	0.79 (0.37–1.21)	0.2
D-Dimer *ng/ml*	147.4 (108.0–226.5)	139.3 (113.4–177.7)	0.8
Insulin *mU/l*	7.35 (5.17–9.14)	6.04 (5.03–10.21)	0.4
Serum Amyloid A** *mg/l*	5.1 (5.1–10.3)	5.1 (5.1–8.4)	*ntp*
**Anti-inflammatory**			
Total adiponectin *µg/ml*	3.61 (2.07–5.86)	2.54 (1.48–3.61)	0.07
HMW adinopectin *µg/ml*	1.06 (0.86–2.46)	0.92 (0.69–1.51)	0.7
IL-27** *pg/mL*	1325 (638–2872)	1111 (430–2287)	0.6
IL-10** *pg/mL*	0.94 (0.69–1.55)	0.65 (0.41–0.82)	0.03
**Chemokines**			
ICAM-1 pg/mL	128589 (10010–145134)	121905 (100908–128912)	0.6
I-TAC pg/mL	257.5 (108.8–339.8)	254.7 (153.7–350.6)	0.9
IP-10 pg/mL	214.1 (168.8–354.7)	237.4 (164.3–415.8)	0.6
Fractalin** pg/mL	82.1 (11.2–415.3)	41.4 (11.2–264.1)	0.5
VCAM-1 pg/mL	129635 (121514–189670)	141891 (117047–164106)	0.6
Mig pg/mL	376 (270–797)	261 (152–794)	0.3
MCP-1 pg/mL	76.9 (31.6–103.4)	51.7 (35.3–77.5)	0.2
E-selectin pg/mL	10344 (8131–33045)	11787 (4928–23219)	0.7
P-selectin pg/mL	125138 (87195–164171)	144574 (92185–189056)	0.7
Soluble CD14 ng/ml	1066 (935–1365)	1043 (885–1281)	0.9

All patients were male, non smokers.

Medians (25th and 75th %tiles) were adjusted by age, hypertension, and duration of antiretroviral therapy using quantile regression.

*Significance determined using a two-tailed t-test based on estimated variance.

**Thresholds for biochemical markers were included in the summary statistic and calculated using the median between the commercial threshold given and 0.

D – detectable; UD – undetectable; us-CRP – ultra-sensitive C-reactive protein; IL – interleukin; HMW – high molecular weight; ICAM-1 – intercellular adhesion molecule-1; I-TAC – interferon inducible T-cell alpha chemoattractant; IP-10 – inducible protein-10; VCAM-1 – vascular cell adhesion molecule-1; MCP-1 – monocyte chemoattractant protein-1.

### Carotid-IMT and residual viremia

As shown in Table 3, mean total c-IMT was significantly different between D and UD groups in univariate analysis. However, after adjusting on age and HOMA-IR (both of which were independently associated with total c-IMT), there was no significant difference between viremia groups (mean±SE, D: 0.768 mm±0.014 versus UD: 0.746 mm±0.009, *p* = 0.2). In further analysis, the difference between viremia groups remained non-significant after separately and additionally adjusting (to the full multivariable model) for years undergoing ART (Δ = 0.024 mm, *p* = 0.14), years of known HIV-infection (Δ = 0.022 mm, *p* = 0.19), years of undetectable HIV-viremia from most recent HIV-VL >50 copies/mL (Δ = 0.021 mm, *p* = 0.2), nadir CD4+ cell count (Δ = 0.028, *p* = 0.11), or protease inhibitor (PI)-use (Δ = 0.022 mm, *p* = 0.2). The following CVD risk factors were also added separately to the full multivariable model, showing no significant differences between viremia groups: HDL- (Δ = 0.021 mm, *p* = 0.2) or LDL-cholesterol (Δ = 0.022 mm, *p* = 0.2), statin-use (Δ = 0.019, *p* = 0.3), and family history of CVD (Δ = 0.023, *p* = 0.18).

**Table 3:** Total and common carotid artery intima media thicknesses between viremia groups.

	Total c-IMT				cca-IMT			
	Univariate		Multivariable		Univariate		Multivariable	
	Δ* (95% CI)	*p*	Δ* (95% CI)	*p*	Δ* (95% CI)	*p*	Δ* (95% CI)	*p*
D- vs UD-viremia groups	0.045 (0.002, 0.089)	0.04	0.021 (−0.012, 0.055)	0.2	0.074 (0.026, 0.123)	0.003	0.059 (0.022, 0.095)	0.002
**Cardiovascular risk-factors**								
Age (per 10 years)	0.077 (0.047, 0.106)	<0.001	0.061 (0.032, 0.089)	<0.001	0.077 (0.040, 0.114)	<0.001	0.048 (0.018, 0.078)	0.002
SBP (per 10 mmHg)	0.015 (0.000, 0.030)	0.06			0.020 (0.003, 0.038)	0.02	0.012 (−0.002, 0.027)	0.09
Hypertension**	0.010 (0.020, 0.179)	0.02			0.166 (0.080, 0.252)	<0.001		
HDL-cholesterol (per mmol/L)	−0.046 (−0.123, 0.031)	0.2			−0.027 (−0.117, 0.064)	0.6		
LDL-cholesterol (per mmol/L)	0.027 (0.005, 0.048)	0.02			0.024 (−0.002, 0.050)	0.07		
Family history of CVD	0.000 (−0.084, 0.086)	0.9			0.045 (−0.053, 0.143)	0.4		
HOMA-IR	0.036 (0.017, 0.055)	0.001	0.025 (0.009, 0.042)	0.003	0.051 (0.031, 0.072)	<0.001	0.034 (0.014, 0.053)	0.001
Statin-use	−0.006 (−0.091, 0.079)	0.9			0.061 (−0.036, 0.159)	0.2		
**HIV-related factors**								
Nadir CD4+ (per 100 cells/mm^3^)	−0.004 (−0.015, 0.007)	0.5			−0.001 (−0.014, 0.012)	0.9		
CD4+ (per 100 cells/mm^3^)	0.004 (−0.007, 0.015)	0.4			0.003 (−0.010, 0.016)	0.7		
Duration of ART (per year)	0.012 (0.005, 0.020)	0.002			0.007 (−0.003, 0.017)	0.15		
Yrs from last HIV VL >50 cp/mL	−0.001 (−0.009, 0.006)	0.7			0.000 (−0.009, 0.009)	0.9		
PI-use	0.025 (−0.016, 0.066)	0.2			0.020 (−0.028, 0.068)	0.4		
Abacavir-use	−0.017 (−0.063, 0.029)	0.5			−0.023 (−0.076, 0.031)	0.4		

All patients were male, non smokers.

*Difference in intima media maximal thickness between groups.

**Hypertension was defined by prior physician‘s diagnosis or systolic blood pressure ≥140 mmHg or diastolic blood pressure ≥90 mmHg.

c-IMT – carotid intima media thickness; cca-IMT – common carotid artery intima media thickness; D – detectable; UD – undetectable; SBP – systolic blood pressure; HDL – high-density lipid; LDL – low-density lipid; CVD – cardiovascular disease; HOMA-IR – homeostatic model assessment of insulin resistance; ART – antiretroviral therapy; VL – viral load; PI – protease inhibitors.

In contrast, a significant difference between groups was found in mean adjusted cca-IMT (mean±SE, D: 0.727 mm±0.015 versus UD: 0.668 mm±0.010, *p* = 0.002), which remained independently associated with age and HOMA-IR. This difference remained statistically significant after separately and additionally adjusting (to the full multivariable model) for years undergoing ART (Δ = 0.059 mm, *p* = 0.003), years of known HIV-infection (Δ = 0.058 mm, *p* = 0.003), years of undetectable HIV-viremia from most recent HIV-VL >50 copies/mL (Δ = 0.059 mm, *p* = 0.002), nadir CD4+ cell count (Δ = 0.059, *p* = 0.002) and protease inhibitor (PI)-use (Δ = 0.060 mm, *p* = 0.002). The significant association continued to remain after additionally adjusting for HDL- (Δ = 0.057 mm, *p* = 0.003) or LDL-cholesterol (Δ = 0.058 mm, *p* = 0.003), statin-use (Δ = 0.042, *p* = 0.04), and family history of CVD (Δ = 0.057, *p* = 0.004).

As shown in Table 4, only age was significantly associated with increased bifurcation c-IMT, while no significant difference was observed between viremia groups (mean±SE, D: 0.840 mm±0.024 versus UD: 0.841 mm±0.016, *p* = 0.9). Conversely, HIV-RNA viremia >1 copy/mL was the only significant variable associated with differences in ica-IMT in multivariable analysis (mean±SE, D: 0.758 mm±0.019 versus UD: 0.707 mm±0.012, *p* = 0.03).

**Table 4:** Bifurcation and internal carotid artery intima media thicknesses between viremia groups.

	Bifurcation IMT				ica-IMT			
	Univariate		Multivariable		Univariate		Multivariable	
	Δ* (95% CI)	*p*	Δ* (95% CI)	*p*	Δ* (95% CI)	*p*	Δ* (95% CI)	*p*
D- vs UD-viremia groups	0.033 (−0.036, 0.101)	0.3	−0.001 (−0.058, 0.059)	0.9	0.051(0.007, 0.096)	0.03	**	
**Cardiovascular risk-factors**								
Age (per 10 years)	0.110 (0.064, 0.157)	<0.001	0.111 (0.062, 0.159)	<0.001	0.032 (−0.006, 0.069)	0.09		
SBP (per 10 mmHg)	0.018 (−0.005, 0.042)	0.13			−0.005 (−0.021, 0.011)	0.5		
Hypertension***	0.070 (−0.057, 0.197)	0.3			0.062 (−0.023, 0.146)	0.15		
HDL-cholesterol (per mmol/L)	−0.068 (−0.185, 0.048)	0.2			−0.036 (−0.116, 0.043)	0.4		
LDL-cholesterol (per mmol/L)	0.043 (0.011, 0.075)	0.01			0.013 (−0.011, 0.036)	0.3		
Family history of CVD	−0.014 (−0.143, 0.115)	0.8			−0.044 (−0.130, 0.041)	0.3		
HOMA-IR	0.042 (0.011, 0.073)	0.009			0.005 (−0.018, 0.027)	0.7		
Statin-use	−0.067 (−0.194, 0.061)	0.3			−0.022 (−0.108, 0.065)	0.6		
**HIV-related factors**								
Nadir CD4+ (per 100 cells/mm^3^)	−0.005 (−0.022, 0.012)	0.5			−0.004 (−0.016, 0.007)	0.5		
CD4+ (per 100 cells/mm^3^)	0.163 (−0.260, 0.586)	0.4			0.080 (−0.212, 0.371)	0.6		
Duration of ART (per year)	0.020 (0.008, 0.031)	0.001			0.008 (0.000, 0.017)	0.046		
Yrs from last HIV VL >50 cp/mL	−0.003 (−0.015, 0.008)	0.6			0.001 (−0.007, 0.008)	0.9		
PI-use	0.022 (−0.041, 0.085)	0.5			0.028 (−0.015, 0.071)	0.2		
Abacavir-use	−0.036 (−0.106, 0.034)	0.3			0.008 (−0.041, 0.057)	0.7		

All patients were male, non smokers.

*Difference in intima media maximal thickness between groups.

**D-viremia was the only significant predictor. Adding duration of ART to the model did not improve model fit (difference in BIC: 0.616) and was hence not included.

***Hypertension was defined by prior physician‘s diagnosis or systolic blood pressure ≥140 mmHg or diastolic blood pressure ≥90 mmHg.

IMT – intima media thickness; ica-IMT – internal carotid artery intima media thickness; D – detectable; UD – undetectable; SBP – systolic blood pressure; HDL – high-density lipid; LDL – low-density lipid; CVD – cardiovascular disease; HOMA-IR – homeostatic model assessment of insulin resistance; ART – antiretroviral therapy; VL – viral load; PI – protease inhibitors.

### Plaque prevalence and residual virema

Plaque prevalence tended to be higher in patients with respective D versus UD-viremia (50.0% vs 24.2%, *p* = 0.1). As expected, plaque was mostly located on the carotid bifurcation segment (*n* = 12) or on several sites (ica and bifurcation, *n* = 2; cca and bifurcation, *n* = 1).

## Discussion

In this highly-selected population, we report for the first time that residual viremia was strongly associated with increased IMT on the common carotid artery segment, broadening similar findings at higher HIV-RNA levels during ART [11] and among patients displaying elite control of HIV-replication. [12], [13] When examining the other carotid artery segments, we observed that HIV-VL between 1-20 copies was the only significant predictor of thicker internal carotid artery IMT. Nevertheless, it was clear that IMT at the bifurcation segment, which is known to be rather heterogeneous between individuals [14], showed the highest variability of all IMT segments measured with high prevalence of plaque. This could help explain why no difference in total c-IMT was observed between viremia groups.

Furthermore, the magnitude of difference observed on the cca-IMT could be considered clinically relevant. For instance, Ando et al. [15] reported in a large cross-sectional study that an average increase in cca-IMT of 0.04–0.06 mm was associated with a 10-year increase in age. Among younger HIV-infected patients, mean cca-IMT was shown to be 0.04 mm higher in patients 40–47 compared to 24–35 years of age. [16] It would then appear that the difference of roughly 0.06 mm between viremia groups, even after adjusting for age, systolic blood pressure, and HOMA-IR, falls in line with clinically important thresholds. Interestingly, preliminary evidence has pointed to a roughly 4% increased absolute difference in prevalent 5-year mortality when comparing patients with 1–19 copies/mL and <1 copy/mL [6], suggesting a potential clinical association with residual viremia. Naturally, these results need to be confirmed in larger epidemiological studies.

Although a wide variety of biomarkers have produced strong associations with higher levels of HIV-replication [6], [17], [18], several studies have failed to demonstrate their significance at <50 copies/mL. [3], [5] From our data, it is hard to determine what role inflammation has on increased c-IMT. The generally higher inflammatory levels observed in patients with detectable versus undetectable HIV viremia indicates that the degree of systemic inflammation may be slightly attenuated, compared to inflammation at higher HIV levels. Larger populations would be needed to properly establish any meaningful difference. Even so, the biological source of inflammation during persistent HIV-viremia remains unclear, as evidence has reported a number of putative mechanisms related to continual production of HIV-RNA from latent reservoirs. [1], [3], [19]

No association with c-IMT and any specific antiretroviral treatment was noted in our study, contrary to other epidemiological reports. [11], [12], [18] Our study sample does have certain obvious differences compared to these studies in that the patient numbers were much smaller and CVD-risk much lower, greatly affecting the power necessary to establish a difference in c-IMT for any one treatment. Nevertheless, future research should pay particular attention to some of the more recent and commonly-used treatments. For example, treatment-experienced patients with well-controlled HIV-RNA have shown substantial reductions in inflammatory markers and markers of monocyte activation after switching to ART containing the integrase inhibitor raltegravir (RAL). [20], [21] Whether these results directly translate into changes in subclinical markers of atherosclerosis is debatable, given that switching to RAL-based regimens failed to show improvement in flow-mediated dilation. [22] Moreover, studies examining both switching to and intensification of RAL-based ART have not demonstrated any further decrease in ultrasensitive HIV-RNA detection [23], [24], leaving it difficult to hypothesize how residual viremia would fit into the association between RAL and inflammation.

One of the major limitations of our study was the few numbers of patients included. We attempted to mitigate the impact of this problem by accounting for two major classical CVD risk factors, smoking and gender, in the study design, where otherwise large variability would have been introduced. As a result, IMT levels were much lower than what has been previously reported among HIV-infected subjects. Indeed, generalizability of our results was limited, yet with the added advantage of increased validity.

Another limitation of our study was that we did not perform repeated measurements of ultrasensitive HIV-RNA, since the aliquots of plasma required to do so exceeded the available amount. Nevertheless, we examined ultrasensitive VL 12-months prior to study visit in a subset of patients (N = 37), showing a proportion of patients with HIV-VL below detection threshold at both time points (45.9%) that was roughly similar to a longitudinal study measuring ultrasensitive HIV-RNA in quadruplicate. [3] Finally, outliers could account for some of the differences observed. We repeated the analysis using robust linear regression, which assigns a lower weight to values with higher absolute residuals, to reconstruct the multivariable models, giving similar models with the same conclusion with respect to all c-IMT segments (data not shown). Furthermore, we employed a multivariable modeling strategy known to limit the number of potential confounders, hence all models could only include a handful of confounders at a time. Future research needs to establish how other CVD risk-factors could play a role in the purported association between residual viremia and increased IMT.

In conclusion, higher IMT in certain carotid artery segments was observed in patients with residual viremia. This finding warrants larger longitudinal studies in order to confirm our results and determine if changes in residual viremia are correlated with changes in inflammatory markers and c-IMT.
